# Stress Cardiomyopathy Following Thoracostomy Tube Placement and Hemothorax Drainage: A Case Report

**DOI:** 10.7759/cureus.45733

**Published:** 2023-09-21

**Authors:** Brandon Kuley, Jeremy J Webb

**Affiliations:** 1 Emergency Medicine, LewisGale Medical Center, Salem, USA; 2 Medicine, Edward Via College of Osteopathic Medicine, Blacksburg, USA

**Keywords:** stress-induced cardiomyopathy, chest wall trauma, thoracostomy tube, post-traumatic hemothorax, tako-tsubo cardiomyopathy (ttc)

## Abstract

Stress cardiomyopathy (SCM) is a clinical phenomenon presenting symptoms suggestive of acute coronary syndrome and defined by acute, but transient, electrocardiogram (ECG) changes and left ventricular wall motion abnormalities. However, no obstructive coronary lesion is identified on catheterization, and pathognomic echocardiogram findings are typically encountered. Multiple causes have been posited in the literature (e.g., severe stress, anxiety, pain, comorbid illness, trauma). We present the case of a 46-year-old female who presented to the emergency department (ED) for delayed left-sided hemothorax (six weeks following a high-speed motor vehicle collision) and developed an acute SCM following large-bore chest tube placement. To our knowledge, no prior cases have been reported immediately following thoracostomy tube placement and hemothorax drainage in the ED setting. We explore possible mechanistic explanations related to our case, which adds to the existing literature on the subject.

## Introduction

Stress cardiomyopathy (SCM) is defined as a transient decrease in cardiac function in the setting of an acute stressor without evidence of an obstructive coronary lesion. The clinical entity was first described in 1990 in a seminal paper by Satoh et al. [1]. Patients may present to the emergency department (ED) with symptoms including chest pain and dyspnea, with an initial workup revealing ST-segment elevation on electrocardiogram (ECG) and a rise in cardiac troponin levels that mimic an acute myocardial infarction. Follow-up coronary catheterization is negative for an obstructive coronary lesion, and the echocardiogram classically shows a “takotsubo” or Japanese octopus-trap appearance with regional wall motion abnormalities of the left ventricle with apical ballooning. It has been surmised that 1-2% of patients presenting with symptoms consistent with acute coronary syndrome are ultimately diagnosed with SCM [2].

Precipitators include physical stress (e.g., pain, infection, respiratory distress) and emotional stress (e.g., grief, panic, interpersonal conflict) [3]. There have also been case reports of SCM developing after trauma. Cases include multisystem blunt trauma related to MVC, severe burns, and isolated chest wall trauma [4-6]. However, there are no reports describing the development of SCM immediately following therapeutic thoracostomy tube placement and hemothorax drainage in the ED setting.

## Case presentation

A 46-year-old female presented to the ED with a cough and worsening dyspnea six weeks after a high-speed motor vehicle collision. On her index visit at an outside hospital, she was diagnosed with multiple left-sided rib fractures, which were treated conservatively, and discharged home. The patient described the subsequent development of a semi-productive cough and worsening dyspnea with minimal exertion. She denied new or worsening chest pain, fever, hemoptysis, interval trauma, or lower extremity swelling and had no prior cardiopulmonary risk factors or disease history.

**Figure 1:**
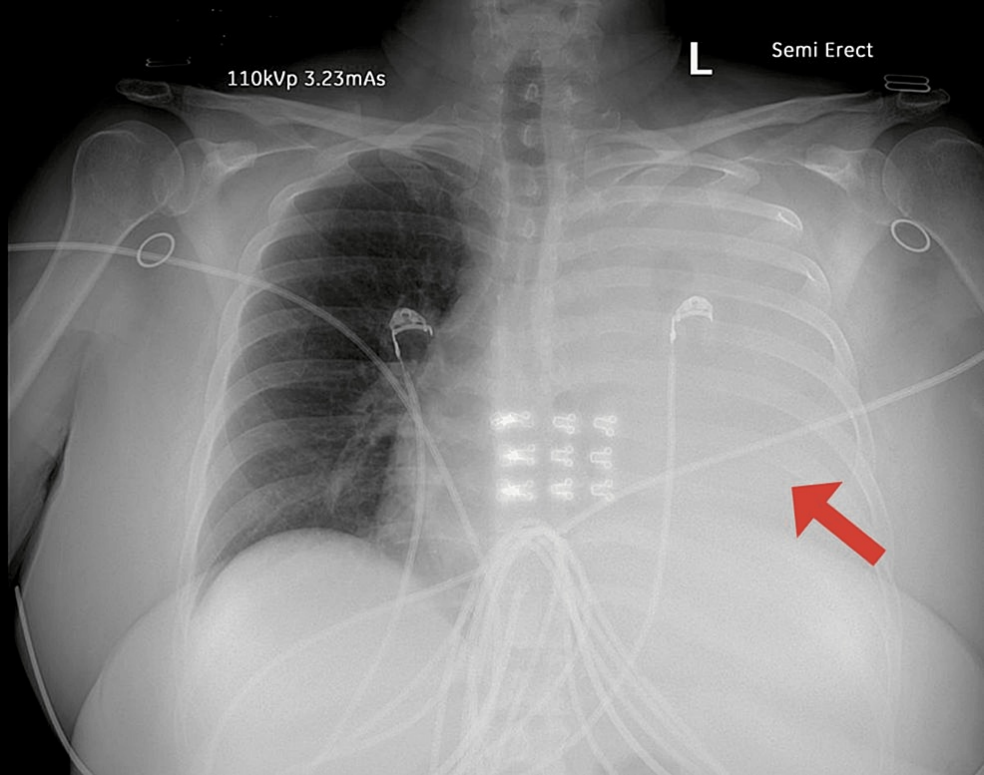
Portable chest radiograph on initial ED presentation showing complete opacification of the left hemithorax.

**Figure 2:**
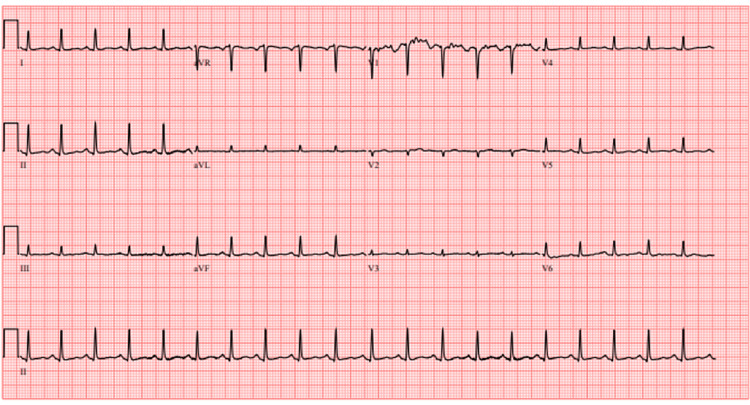
Initial ECG prior to thoracostomy tube insertion.

Physical examination revealed reduced lung sounds on the left side and tenderness along the lower left chest wall. The patient’s vital signs were significant for a heart rate of 126 beats per minute and a respiratory rate of 27 breaths per minute. Her initial BP was 151/71, oxygen saturation remained at 99% on room air at rest, and her temperature was 36.7 °C. Here initial laboratory studies can be found in Table 1. Portable chest radiography revealed a complete left-sided hemothorax with mediastinal shift (Figure 1). Subsequent computed tomography (CT) of the chest without contrast confirmed x-ray findings and demonstrated subacute fractures of the left sixth through eighth posterior ribs and a trace pericardial effusion. Initial ECG revealed sinus tachycardia, with poor R wave progression in anterior leads, but did not show evidence of ST-segment changes (Figure 2). Preparations were made for thoracostomy tube placement and evacuation of the hemothorax.

**Table 1:** Patient's initial lab parameters and facility's reference range.

Lab Test	Patient’s Lab Parameters	Reference Range
Hemoglobin	10.4 g/dL	13-17.3 g/dL
High Sensitivity Troponin-T	< 2.6 ng/L	< 53.48 ng/L
D-Dimer	1,249 ng/mL	< 500 ng/mL

The patient underwent conscious sedation with 75 mcg of fentanyl and 2 mg of midazolam, and 2% lidocaine with epinephrine was used for local anesthesia at the site of insertion. Additional analgesia and sedation with 4 mg of morphine and 50 mg of ketamine were provided during the procedure. A 28-French chest tube was inserted at the anterior axillary line in the left fourth intercostal space. The tube was secured with silk suture and attached to standard water seal and suction. Nearly two liters of mixed serosanguinous output was collected.

**Figure 3:**
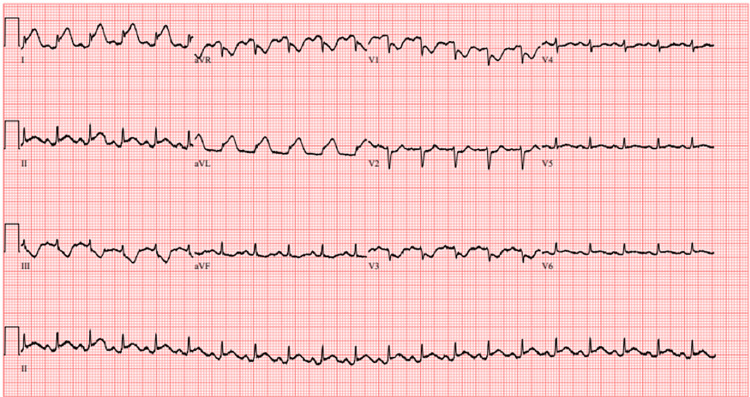
ECG after thoracostomy tube placement.

**Figure 4:**
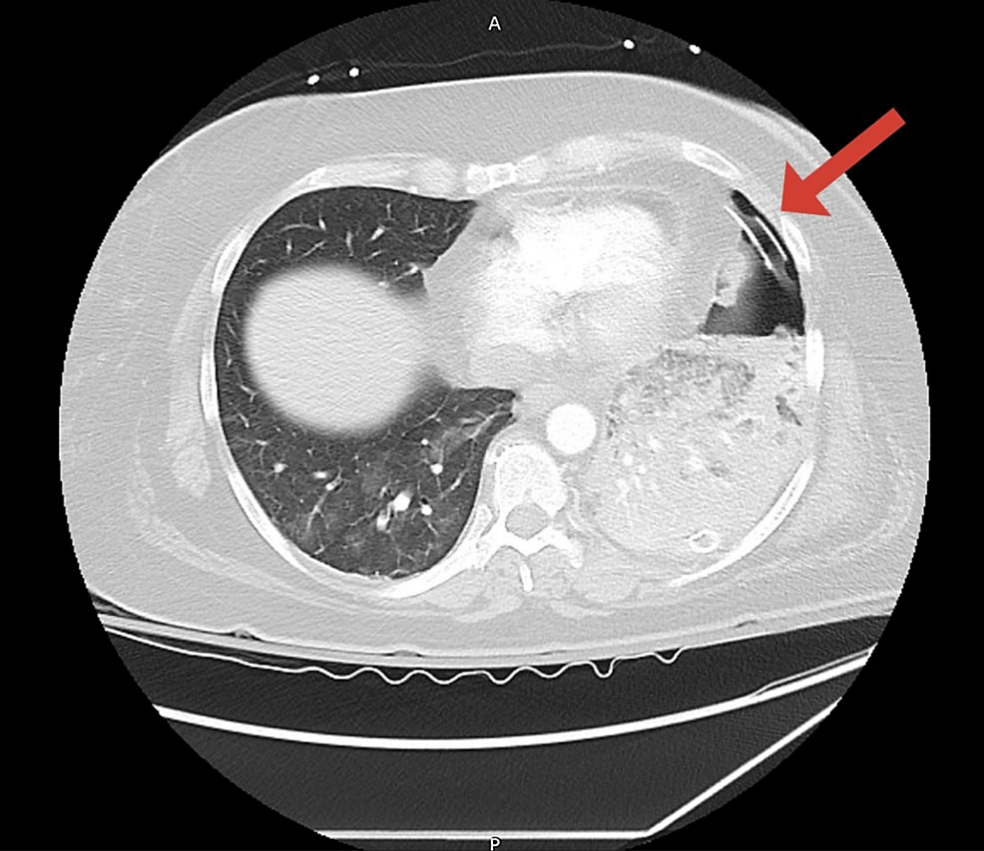
Chest CT revealing the location of the thoracostomy tube tip adjacent to the cardiac apex.

**Figure 5:**
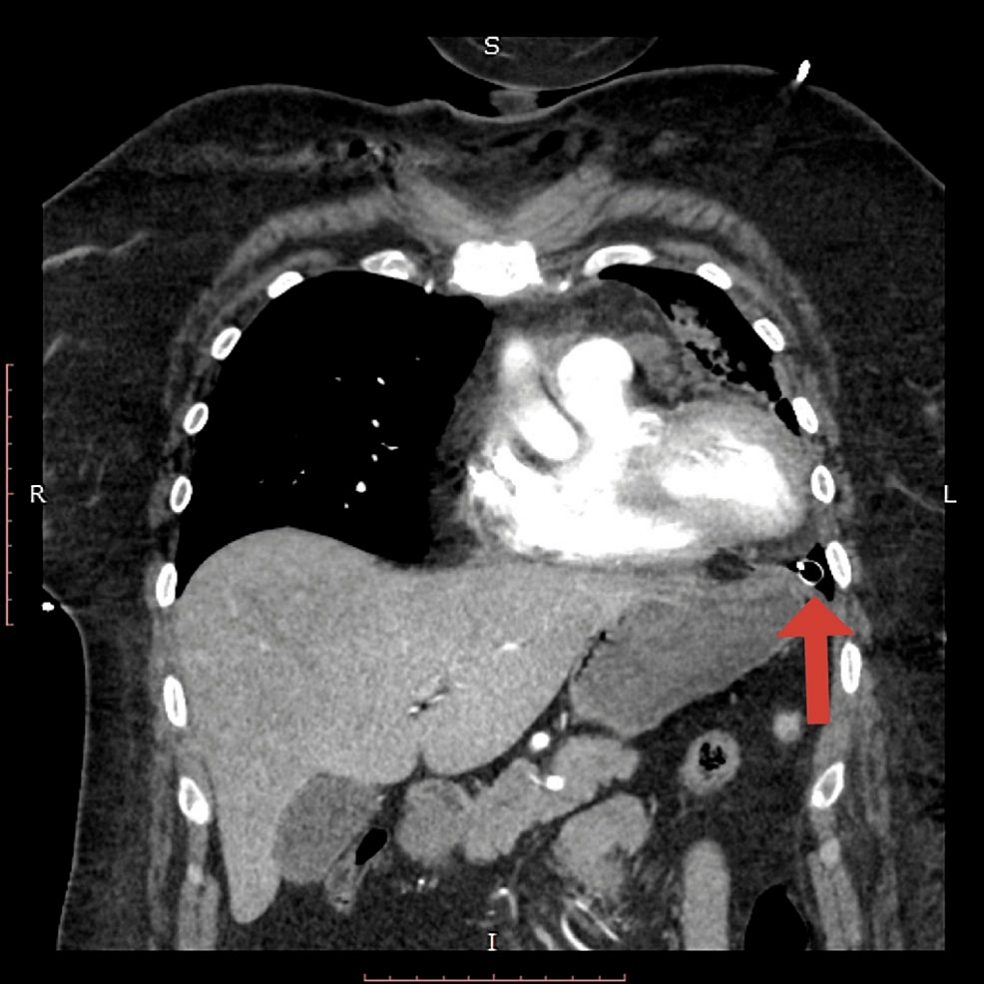
Coronal view CT showing the location of the chest tube inferior to the cardiac apex.

Upon completion of the procedure, ST-segment changes were noted on the monitor, and a repeat ECG was performed (Figure 3). The patient denied chest pain but soon developed hypoxia with an oxygen saturation of 83% on room air, which improved to 93% on a 15-L non-rebreather mask. Immediate follow-up CT angiography of the chest did not show pulmonary embolism, vascular injury, vascular dissection, or active bleeding. Lung re-expansion was noted with diffuse alveolar infiltrate consistent with pulmonary edema. The course of the thoracostomy tube was deemed appropriate; however, the tip was noted to lie in close proximity to the inferior cardiac apex (Figures 4, 5).

Within two hours, the ECG changes had resolved spontaneously. The patient remained chest pain-free, and supplemental oxygen was able to be weaned in the ED. High-sensitivity troponin T peaked at 5,956 ng/L. The patient was given 324 mg of aspirin and was eventually cleared for anticoagulation by the trauma surgery team. The patient was admitted to the intensive care unit and later taken for coronary catheterization, which did not reveal any significant coronary disease, acute obstructive coronary lesions, or significant alterations in coronary anatomy. Left ventriculography was interpreted as "anterior apical akinesis." Subsequent echocardiography revealed severe anteroapical hypokinesis with a left ventricular ejection fraction of 40-45% (Video 1). She was ultimately diagnosed with SCM by the treating cardiologist, initiated on beta-blocker therapy, and discharged from hospital after hemothorax resolution and chest tube discontinuation. No follow-up echocardiography has been performed to date.

**Video 1:** Patient's inpatient echocardiogram. Colorized B-mode, zoomed apical-4 chamber view.

**Figure 6:**
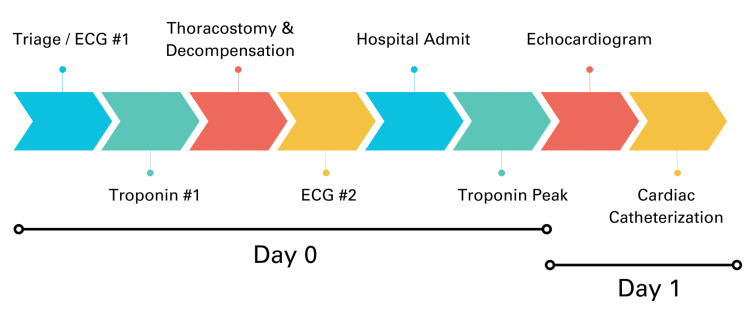
Timeline of major ED and inpatient events from triage to cardiac catheterization. Day 0 refers to the day of the initial presentation to the ED. Day 1 refers to the first inpatient hospital day.

A summary timeline of hospital events, from triage to cardiac catheterization, can be found in Figure 6 below.

## Discussion

The above case illustrates a potential unique cause for SCM in the ED setting. Our patient's presentation was characterized by acute ST-segment elevation in the setting of acute hypoxia and dyspnea following thoracostomy tube placement for large-volume hemothorax. Subsequent cardiac catheterization showed no obstruction, and the echocardiogram was concerning for SCM.

International consensus diagnostic criteria for SCM/Takotsubo's Cardiomyopathy can be found in Table 2.

**Table 2:** International consensus diagnostic criteria. Adapted from Ghadri et al. [7].

International Takotsubo Diagnostic Criteria
Patients show transient left ventricular dysfunction (hypokinesia, akinesia, or dyskinesia) presenting as apical ballooning or midventricular, basal, or focal wall motion abnormalities. Right ventricular involvement can be present. Besides these regional wall motion patterns, transitions between all types can exist. The regional wall motion abnormality usually extends beyond a single epicardial vascular distribution; however, rare cases can exist where the regional wall motion abnormality is present in the subtended myocardial territory of a single coronary artery (focal TTS).
An emotional, physical, or combined trigger can precede the Takotsubo syndrome event, but this is not obligatory.
Neurologic disorders (e.g. subarachnoid hemorrhage, stroke/transient ischemic attack, or seizures) as well as pheochromocytoma may serve as triggers for Takotsubo syndrome.
New ECG abnormalities are present (ST-segment elevation, ST-segment depression, T-wave inversion, and QTc prolongation); however, rare cases exist without any ECG changes.
Levels of cardiac biomarkers (troponin and creatine kinase) are moderately elevated in most cases; significant elevation of brain natriuretic peptide is common.
Significant coronary artery disease is not a contradiction in Takotsubo syndrome.
Patients have no evidence of infectious myocarditis.
Postmenopausal women are predominantly affected.

ECG findings of ST elevation are seen in nearly half of all cases of SCM, typically in the anterior leads, and cardiac biomarkers are frequently elevated [3,8]. Echocardiogram at presentation often demonstrates reduced left ventricular function with regional wall motion abnormality, and cardiac catheterization reveals the absence of a culprit coronary lesion. While the prognosis for these patients is favorable, as most make a full recovery, in-hospital mortality has been estimated at 5% [8]. Rare complications include intra-cardiac thrombus formation and cardiogenic shock [9]. Patients who survive the acute episode typically recover systolic ventricular function in one to six weeks [8]. Our patient's clinical course in-hospital was uncomplicated. There has been no follow-up to date. 

Acute psychological and/or physical stressors are often associated with the development of this condition. Post-procedurally, SCM has been documented during non-cardiac surgeries, such as tracheotomy, cholecystectomy, lung biopsy, and colectomy [10]. However, no cases have been reported immediately following therapeutic thoracostomy tube placement in the ED setting. Stressor-induced peripheral sympathetic nerve activation, catecholamine surge, vasoconstriction, and myocardial toxicity/stunning have been suggested as potential pathophysiological mechanisms [9-11]. In this case, we identify several potential triggers. These include possible inadequate analgesia and sedation during a painful procedure, transient post-procedural hypoxia related to large volume evacuation and re-expansion pulmonary edema with subsequent ventilation-perfusion mismatch and shunting, and local pericardial irritation from chest tube tip position near the cardiac apex. This last point may have been amended by insertion technique or tube repositioning/withdrawal.

The differential diagnosis in this case also included thrombotic ST-elevation myocardial infarction with spontaneous reperfusion as well as coronary vasospasm. These were felt to be less likely by the treating cardiology team after a review of her catheterization and echocardiogram results. However, it is important to rule out acute coronary syndrome in these scenarios as the two clinical entities present similarly.

This case demonstrates that thoracostomy tube placement and hemothorax drainage may provoke an acute SCM. It is a reminder that even therapeutic non-cardiac procedures may precipitate the syndrome.

## Conclusions

This case outlines a unique cause of SCM following thoracostomy tube placement and hemothorax drainage in the ED setting. At the time of presentation, the ECG, echocardiographic, and laboratory findings may be significantly abnormal in the patient with SCM. It is important to rule out other life-threatening causes, which may present in a similar fashion (e.g., acute coronary syndrome and myocardial infarction). Fortunately, most cases of SCM will be transient and improve spontaneously without long-term sequelae. Increased awareness of the potential to provoke SCM in the ED or inpatient ward may aid in future prevention and management.

## References

[1] Satoh H, Tateishi H, Uchida T (1990). Takotsubo-type cardiomyopathy due to multivessel spasm. Clinical Aspect of Myocardial Injury: From Ischemia to Heart Failure.

[2] Akashi YJ, Goldstein DS, Barbaro G, Ueyama T (2008). Takotsubo cardiomyopathy: a new form of acute, reversible heart failure. Circulation.

[3] Templin C, Ghadri JR, Diekmann J (2015). Clinical features and outcomes of takotsubo (stress) cardiomyopathy. N Engl J Med.

[4] Cimaroli S, Maniar Y, Ciancarelli J, Stright A, Joseph D (2023). Takotsubo cardiomyopathy following blunt trauma: early recognition and diagnosis. Trauma Case Rep.

[5] Ahmed Y, Rafique M, Ahmad S, Omar B, Malozzi C (2022). Reverse takotsubo cardiomyopathy in a patient with commotio cordis. J Med Cases.

[6] Kigure R, Akiyama G, Tosa M, Ogawa R (2023). A case of takotsubo cardiomyopathy after dermal burn on the face and hands. Plast Reconstr Surg Glob Open.

[7] Ghadri JR, Wittstein IS, Prasad A (2018). International expert consensus document on Takotsubo syndrome (Part I): clinical characteristics, diagnostic criteria, and pathophysiology. Eur Heart J.

[8] Medina de Chazal H, Del Buono MG, Keyser-Marcus L, Ma L, Moeller FG, Berrocal D, Abbate A (2018). Stress cardiomyopathy diagnosis and treatment: JACC state-of-the-art review. J Am Coll Cardiol.

[9] Akhtar MM, Cammann VL, Templin C, Ghadri JR, Lüscher TF (2023). Takotsubo syndrome: getting closer to its causes. Cardiovasc Res.

[10] Tsuchihashi K, Ueshima K, Uchida T (2001). Transient left ventricular apical ballooning without coronary artery stenosis: a novel heart syndrome mimicking acute myocardial infarction. J Am Coll Cardiol.

[11] Paur H, Wright PT, Sikkel MB (2012). High levels of circulating epinephrine trigger apical cardiodepression in a β2-adrenergic receptor/Gi-dependent manner: a new model of Takotsubo cardiomyopathy. Circulation.

